# Antibacterial efficacy of silver nanoparticles (AgNPs) against metallo-β-lactamase and extended spectrum β-lactamase producing clinically procured isolates of *Pseudomonas aeruginosa*

**DOI:** 10.1038/s41598-022-24531-9

**Published:** 2022-11-30

**Authors:** Maria Muddassir, Almas Raza, Sadaf Munir, Ahmad Basirat, Muddassir Ahmed, Mazia Shahid Butt, Omair Arshad Dar, Syed Shoaib Ahmed, Saba Shamim, Syed Zeeshan Haider Naqvi

**Affiliations:** 1 Institute of Molecular Biology and Biotechnology (IMBB), The University of Lahore, Defence Road Campus, Lahore, Pakistan; 2 Combined Military Hospital, Lahore, Pakistan; 3 King Edward Medical University, Lahore, Pakistan; 4 Social Security Hospital, Gujranwala, Pakistan; 5 Lahore General Hospital, Lahore, Pakistan; 6 Pak Emirates Military Hospital, Rawalpindi, Pakistan; 7 Al-Aleem Centre for Advanced Studies and Research, Gulab Devi Educational Complex, Lahore, Pakistan

**Keywords:** Microbiology, Molecular biology, Nanoscience and technology

## Abstract

Resistance to carbapenems is a global threat, especially in developing countries with limited health resources. Prevalence, antibiogram, PCR detection of antibiotic resistance genes, and potency of Silver Nanoparticles (AgNPs) against multidrug-resistant (MDR) *Pseudomonas aeruginosa* were studied. Kirby-Bauer disc method and PCR were used to study antibiogram and drug resistance genes respectively in 255 isolates of *Pseudomonas aeruginosa* obtained from a tertiary care hospital. Silver nitrate (AgNO_3_) precursor salts were reacted with *Aspergillus flavus* culture filtrate to trigger the extracellular mycosynthesis of AgNPs. Mycosynthesis was first monitored regularly by visible ultraviolet spectroscopy that recorded AgNP peaks of approximately 400–470 nm. Confirmation by Transmission electron micrographs provided confirmation of AgNPs formed within a range of 5–30 nm. Individual and combined antibacterial activity of ten antibiotics and AgNPs was analyzed. Pearson correlation coefficients (r) were calculated for phenotypic and genotypic multidrug resistance. Data were evaluated using SPSS version 20. p-value < 0.05 was considered statistically significant. 61.5% were carbapenemase producers (p < 0.01). The recorded frequency of *bla*_IMP-1_, *bla*_SHV_, *bla*_VIM_, *bla*_OXA_, and *bla*_TEM_ were 13%, 32%, 15%, 21%, and 43%, respectively. The reducing order of antimicrobial activity of antibiotics and AgNPs was piperacillin/tazobactam + AgNPs (31 mm), cefoxitin + AgNPs (30 mm) > amikacin + AgNPs (25 mm) > aztreonam + AgNPs (23 mm) > meropenem + AgNPs (22 mm) > imipenem + AgNPs (20 mm) > gentamycin + AgNPs (17 mm) > ciprofloxacin + AgNPs (16 mm) > cefoperazone/sulbactam + AgNPs (14 mm) ≥ ceftazidime + AgNPs (14 mm). The conjugated effect of AgNPs plus antibiotics showed a 0.15–3.51 (average of 2.09) fold-area augmentation of antimicrobial activity. AgNPs conjugated with antibiotics effectively inhibited MDR *Pseudomonas aeruginosa*. To the best of our understanding, this is an inaugural report from Punjab Pakistan enlisting co-expression of Metallo-β-lactamases, extended-spectrum β-lactamases, and AmpC-β-lactamase plus activity of antibiotic-AgNPs.

## Introduction

Multidrug-resistant *Pseudomonas aeruginosa* (MDR-PA) has emerged as a life-threatening opportunistic pathogen globally in the last two decades particularly in Pakistan, where it has become a severe concern of the health sector becoming a leading cause of nosocomial infections, particularly in patients with postoperative surgical injuries, intensive care units, burn and trauma units, and in those with a pre-existing pulmonary disease such as cystic fibrosis^1,2^. Long-term hospitalization frequently leads to infections by *Pseudomonas aeruginosa* namely malignant external otitis, endocarditis, ophthalmitis, meningitis, septicemia, and pneumonia^1,2^. Multidrug-resistant *Pseudomonas aeruginosa* is by nature resistant to various classes of antimicrobial drugs because of the presence of efflux pumps plus allowing low permeability of the microbial membranes^2^. Centers for Disease Control and Prevention (CDC) reports that more than 32,600 clinical infections in the US are caused by *Pseudomonas aeruginosa,* causing 2700 deaths annually^3^. Metallo-β-lactamase (MBL) enzymes break down a wide variety of β-lactam drugs, counting carbapenems too^4^. According to the Ambler classification of β-lactamase enzymes, they are divided into four classes (A, B, C, D); MBLs belong to class B enzymes, including VIM, IMP, NDM-1, and GIM^4^. Multidrug-resistant *Pseudomonas aeruginosa-*producing Metallo-β-lactamases are causative for various diseases, posing a severe health issue, as resistance against multiple antibiotics is on the rise, especially in Asian countries, including India, Pakistan, and Bangladesh therefore widespread use of antibiotics should be discouraged^5^. Recently, various investigations have reported the emergence of multidrug-resistant bacterial pathogens originating from humans, cattle, birds, and fish increasing the necessity of discovering novel, potent and safe antimicrobials calling for vigorous routine antibiotic susceptibility testing along with the screening of the emerging MDR strains^6,7^. In recent times, the slower improvement of antimicrobial agents has worsened the situation increasing the need of searching for alternative treatment regimes as a substitute for antibiotics^6,7^. A pioneering study reported the presence of 42% *bla*_VIM_ gene multidrug-resistant gram-negative rods^8^. Yet another study from Pakistan reported the presence of *bla*_VIM_ in 12% of multidrug isolates of *Pseudomonas aeruginosa*^9,10^. Algammal *et al*. report that on the basis of antibiogram as well as molecular analysis of the resistance genes, a majority of tested isolates prove to be multi-drug resistant to six major classes of antimicrobials (penicillins, tetracyclines, aminoglycosides, sulfonamides, fluoroquinolones, and lincosamides)^11^. It has been proposed that with an increasing incidence of MDR *Pseudomonas aeruginosa*, newer treatment options such as nanoparticles and other natural products should be explored^12^. Metallic nanoparticles were studied to act as promising alternatives to routine antibiotics for combating and defeating common resistance in pathogens that include target site modification, promoted efflux of drugs via membranes, and enhanced expression of the efflux pumps along with inactivation of enzyme and reduced permeability of the membranes^13,14^. Silver nanoparticles (AgNPs) have been stated as potential agents that have efficacy as antibacterial agents and can help combat nosocomial infections^15^. AgNPs adsorb and penetrate the bacterial cell walls, ultimately leading to the destruction of bacterial cells through the formation of free radicals^13–15^. Additionally, silver nanoparticles can give rise to silver ions^14^. These silver ions can form bonds with crucial organelles and lead to their dysfunction^14–16^. According to WHO, the point prevalence of nosocomial infections ranges from 5.7% in Low-Income and 19.1% in Middle-Income Countries (LMICs) to 3.5–12% in developed nations. The last few years have witnessed an increased interest in the application of Silver Nanoparticles as therapeutic regimens due to low toxicity in the environment, an increased rate of surface capacity, and the ability to inhibit the formation of biofilm that is required for the evasion of pathogens^17^.

The current study is a front-runner report from Pakistan, reporting the simultaneous existence of Metallo-β-lactamase (MBL), extended-spectrum β-lactamase (ESBL), and AmpC drug resistance genes. The current study is also the first to report efficient effects of multiple antibiotic-AgNPs combinations against multidrug-resistant isolates of *Pseudomonas aeruginosa* from Punjab, Pakistan.

## Methodology

### Ethical approval statement

The study was undertaken following ethical approval from the Research Ethics Committee, IMBB The University of Lahore viz Ref # IMBB/UOL/20/138.

### Ethical guidelines and consent to participate

The current study was carried out following appropriate guidelines and regulations followed by informed written consent from all participants.

### Sampling procedures

In total, 255 isolates of *Pseudomonas aeruginosa* obtained from multiple clinical specimens from different departments of a tertiary care hospital were obtained including urine (27.8%), wound swabs (34.9%), sputum (13.7%), pus (7.05%), blood (11.7%), and tissue (4.7%) were processed further. The prevalence of *Pseudomonas aeruginosa* was 22.0% (255/1159). 145 isolates that tested positive for *Pseudomonas aeruginosa* were from females, while 110 were from males.

### Bacterial isolation and identification

The clinical isolates were confirmed by culturing on Pseudomonas cetrimide agar. The morphological characteristics of colonies were used for the identification of the isolates. Identification of isolates for *Pseudomonas aeruginosa* was based on bacterial culture and staining characteristics. Isolates were characterized biochemically using catalase, urease, indole, citrate utilization, lactose, lysine decarboxylation, and glucose fermentation tests^18^. Identification of *Pseudomonas aeruginosa* was by growth on Pseudomonas cetrimide agar plus API20NE identification strips (bioMerieux, France). Identified strains were stored in 30% glycerol broth at − 70 °C. The largest number of isolates were obtained from patients falling in the age group of 40–49 years. Percentage of *Pseudomonas aeruginosa* isolated from departments counted to surgery (36.8%), orthopedics (13.3%), gynecology (7.0%), ICU (11.4%), medicine (25.9%), and ENT (5.5%) (p ≤ 0.001). Specimen-wise isolation was performed with wound swabs (34.9%), tissue (4.7%), blood (11.7%), sputum (13.7%), urine (27.8%), and pus (7.05%). Resistance to imipenem was exhibited by 135 isolates while resistance to ceftazidime was shown by 153 isolates. These isolates were further evaluated employing molecular methodologies.

### Determination of antibiotic susceptibility

Antibiotic sensitivity was studied using the Kirby-Bauer technique to evaluate antibiotic sensitivity of *Pseudomonas aeruginosa* isolates^19^. Following the recommendations of CLSI 2019 (Clinical & Laboratory Standards Institute), sensitivity testing was conducted on Mueller–Hinton agar^20^. The antibiotic discs (bioMérieux, France) specific for studying gram-negative bacteria, including Aminoglycosides (amikacin/AMK 30 µg, gentamicin/GEN 10 µg), Carbapenems (imipenem/IMP 10 µg, meropenem/MEM 10 µg), Monobactams (aztreonam/AZT 10 µg), Penicillins (piperacillin/tazobactam/TZP 100 µg), Gyrase inhibitors (ciprofloxacin/CIP 5 µg), Cephalosporin (ceftazidime/CAZ 30 µg, cefoxitin/CFX 30 µg, cefoperazone/sulbactam/SCF 75–10 µg) were used. The potency of the antibiotic discs was studied using the American Type Culture Collection (ATCC) standard reference strains (*P. aeruginosa* ATCC27853). For studying antimicrobial susceptibility, a 0.5% McFarland turbidity standard was employed for standardizing the bacterial inoculum suspension^21^. The sensitivity test results were utilized to determine the multiple antibiotic resistance index (MAR) of *Pseudomonas aeruginosa* isolates. MAR helps estimate resistance trends against multiple antimicrobial drugs and indicates the emergence of novel resistant bacterial strains. MICs (µg/mL) of meropenem and imipenem plus cefoxitin against multidrug-resistant *Pseudomonas aeruginosa* were determined by E-strip (Thermo Fisher Scientific, UK). A summarization of MIC of used antibiotics has been tabulated (Table 1). In accordance with Magiorakos *et al*., the tested isolates would fall in the category of MDR if the isolates expressed resistance to at least one antimicrobial in three used or more antibiotics^22^.

**Table 1:** Summarization of E-test^®^ interpretive criteria of MIC for *Pseudomonas aeruginosa* in accordance with CLSI 2019^23^.

Antibiotic	Code	Zone Diameter (nearest whole mm) S ≥	Zone Diameter (nearest whole mm) R ≤	*Pseudomonas aeruginosa* ATCC 27853 MIC^a^ µg/mL
Gentamicin	GEN	8	4	1–4
Amikacin	AMK	32	16	1–4
Ciprofloxacin	CIP	2	0.5	0.25–1
Imipenem	IMP	8	2	1–4
Ceftazidime	CAZ	32	8	1–4
Aztreonam	AZT	32	38	2–8
Piperacillin/Tazobactam	TZP	128	16	1–8
Meropenem	MEM	8	2	1–4
Cefoperazone/Sulbactam	SCF	21	15	

^a^Minimun inhibitory concentration measured in µg/mL.

### Phenotypic detection of carbapenemase and metallo-β-lactamases

Carbapenemases were studied following the combined disc synergy test (CDST) and Modified Hodge test (MHT)^4^. Whereas, the production of Metallo-β-lactamases was observed by the combined disc synergy test (CDST) as per the guidelines of CLSI 2019. This test is based on using a disc of imipenem alone and a disc of IMP/EDTA disc (Oxoid, Inc. Canada) according to methodology^4^. A combination disc synergy test (CDST) using a solitary imipenem disc along with an IMP/EDTA disc (Oxoid, Inc. Canada) was performed following the suggested method by Wadekar *et al*.^24^. The modified Hodge test (MHT) was performed following the methods of Kumar *et al*.^25^. Criteria by CLSI 2019 were used to analyze the results. IMP/EDTA E-Strips and IMP alone were used for detecting MBLs as per the manufacturer’s instructions (Liofilchem^®^, Italy)^26^.

### Metallo-β-lactamase: molecular characterization

#### Molecular assays

In accordance with the previously stated method, extraction of the template DNA was performed from isolates^27^. The PCR mixture included 200 µM dNTPs, 50 ng DNA templates, 0.5 U Taq Polymerase, 10 pM primers, 1.5 mM MgCl_2_, and giving an eventual volume of 25 µL. Products of PCR were evaluated and construed for 30 min at 70 V. A 1.5 w/v agarose gel plus 500 µg/100 mL ethidium bromide was used to analyze the products.

#### Detection of MBL and ESBL genes

Confirmation of *Pseudomonas aeruginosa* isolates was done by singleplex PCR. Primer sequences selected for detecting the *bla*_IMP-1_, *bla*_TEM_, *bla*_SHV_, *bla*_OXA_, and *bla*_VIM_ genes have been reported in previous studies^28^. *Pseudomonas aeruginosa* isolates for the *bla*_VIM_ plus *bla*_IMP-1_ genes were screened with the help of singleplex PCR with reported primers^29,30^. The existence of gene *bla*_AmpC_ in *Pseudomonas aeruginosa* was evaluated through PCR amplification of 1063 bp (Table 2). The DNA amplicons were placed in a 1.5% agarose gel for 60 min at 120 V. A UV light trans-illuminator gel documentation system helped visualize the amplified products (Thermo Fisher Scientific, US). Conditions for PCR have been enlisted in Supplementary Table 1 (ST_1).

**Table 2:** Sequence of primers used for detecting MBL^a^, ESBL^b^-type variants and *bla*AmpC.

Primers	Sequence	Annealing Temperature (Tm °C)	Product of PCR	References^c^
*bla*_TEM_	CCCCGAAGAAGTCCTTTC ATCAGCAATAGTCCCAGC	56	500	^31^
*bla*_IMP-1_	AGCGCAGCATATTGATTGC ACAACCAGATGCTGCCTTACC	54	587	^28^
*bla*_SHV_	AGGGCTTGACTGCCATTTTG ATTTGCGTGATTTCATTT	55	400	^31^
*bla*_*AmpC*_	CTTCCACACTGCTGTTCGCC-TTGGCCAGGATCACCAGTCC	66	1063	^32^
*bla*_OXA_	ATATCTCGCTTGTTGCATCTCC AAACCCTTCAGCTCATCC	55	600	^31^
*bla*_VIM_	ATGGTCGTTATGGCATATC TGGGCCGTGTCAGCCAGAT	57	510	^29^

^a^Metallo β-lactamase, ^b^extended spectrum β-lactamase.

^c^Enlisted in references.

### Fungal biomass cultivation

AgNPs myogenesis was carried out employing *Aspergillus flavus*. Aerobic cultivation of fungal biomass was carried out using a liquid medium that contained malt extract in a concentration of 0.3 g/100 mL, glucose 1.0 g/100 mL, yeast extract 0.3 g/100 mL, and peptone 0.5 g/100 mL. Medium’s pH was adjusted initially to be 5.8. Growth of fungal culture was carried out at 28 °C at 150 rpm using an orbital shaker. Extraction of the fungal mass was done through filtration employing Whatman filter paper no 1. Later, this culture helped synthesize nanoparticles.

### Assay for synthesizing nanoparticles

Almost 200 mL of fungal culture without mycelia that contained 0.1 M AgNO_3_ as precursor salt was collected in an Erlenmeyer flask having a volume of 500 mL. This flask was incubated on a shaker at 150 rpm in an ill-lit condition at 28 °C for a duration of 96 h. In accordance with the methods described by Bhainsa *et al*. both positive control i.e. culture filtrate with the exception of silver salt along with negative control, i.e. AgNO_3_ solution was run along with experimental flasks^33^.

### Characterization of AgNPs by transmission electron microscopy and X-ray diffraction

Characterization of myco-genized AgNPs was done by visual observations, Ultraviolet Visible (UV-Vis) Spectrophotometry, XRD, TEM, SEM and DLS. During the assay, 1 mL of sample volume was obtained from the reaction mixture at time intervals of 0, 2, 4, 6, 24, 48, 72 and finally 96 h. Absorbance of sample was recorded at wavelengths 200–800 nm employing a UV–visible spectrophotometer (Agilent 8453 UV–Vis, Agilent Technologies, USA). Colloid suspension from silver containing reaction mixture was eventually concentrated by centrifugation for 20 min at 12,000 rpm (centrifuge Model H-251, Kokusan Co, Ltd, Tokyo, Japan). AgNPs were obtained by washing the silver powder thrice with sterile deionized water and pure ethanol. Thereafter, nanoparticles had to pass the process of microcentrifugation (Microfuge^®^ 18 Centrifuge, Beckman Coulter, USA). Eventually, the removal of the supernatant was performed followed by overnight drying in the oven. AgNPs were finally prepared in powdered form. Nanoparticle comprising dried sample drop-coated films on silica were eventually exposed to analysis by X-ray diffraction (XRD) working in transmission mode at 20 mA, 30 kV with Cu Kα radiation (X’pert PRO XRD, PANalytical BV, The Netherlands). The formation of AgNPs film took place on carbon-coated copper transmission electron microscopy (TEM) grids that were examined by TEM at an 80 kV accelerating voltage (JEM-1010, JEOL Ltd, Tokyo, Japan). Characterization of silver nanoparticles by UV–Vis Spectra, XRD analysis, Size distribution profile of AgNPs, and TEM Micrograph analysis has been discussed in detail by Naqvi *et al*.^30,34^.

### AgNPs antibacterial activity against multidrug-resistant﻿ *Pseudomonas aeruginosa.*

AgNPs were prepared and procured from CRIMM (Centre for Research in Molecular Medicine) at the University of Lahore. To evaluate the antibacterial action of AgNPs, the "disc diffusion method" was employed^35^. Individual and combined antibacterial activities of common antibiotics plus AgNPs were explored against multidrug-resistant isolates of *Pseudomonas aeruginosa* by the Kirby–Bauer disk-diffusion method^13,14^.

### Preparation of bacterial suspension and silver nanoparticles

LB broth (Oxoid, UK) was used to prepare a 0.5 MacFarland suspension of MBL-producing isolates of *Pseudomonas aeruginosa*. A uniform suspension of AgNPs concentrated at 1000 μg/10 mL (stock solution equal to 100 μg/mL) was prepared by dissolving AgNP powder (1 mg) in 10 mL normal saline^36,37^

### Assays for antibacterial activity

The method of disc diffusion was employed for the analysis of the antibacterial efficacy of AgNPs against multiple drug-resistant strains of *Pseudomonas aeruginosa* that were identified from obtained specimens. MIC and MBC values for AgNPs were also evaluated^38^. Nanoparticles of silver nitrate derived from *Aspergillus flavus* were prepared and obtained by the microbiology lab (T-3 and 4), The University of Lahore. Measured weight One milligram of silver nanoparticles was mixed with 10 mL of normal saline for 15 min using a sonicator, and the prepared solution was 100 ppm (1000 µg/10 mL = 100 ppm). The prepared silver NP solution was dropped in amounts of 10, 20, 30, 40, and 50 µL with the help of a pipette on homemade 6 mm discs prepared from plain blotting paper and air-dried for a few seconds. Methodology of disc diffusion method was employed to assess the antibacterial action of AgNPs^13,39^. *Pseudomonas aeruginosa* was streaked on Mueller-Hinton agar for assessment of the antibacterial activity. The Petri plates were incubated at 37 °C for 24 h. Fresh cultures were then used to make the suspension. To maintain the turbidity of the bacterial culture, 0.5% McFarland solution was employed for comparison. One millilitre of this suspension (inoculum) was then added and spread on Mueller-Hinton Agar medium plates. Next, nanoparticles containing air-dried sterile filter paper discs were positioned at suitable distances. Following labelling, the plates were kept at 37 °C for 24 h.

### MIC and MBC of AgNPs (µg/mL)

**Figure 1:**
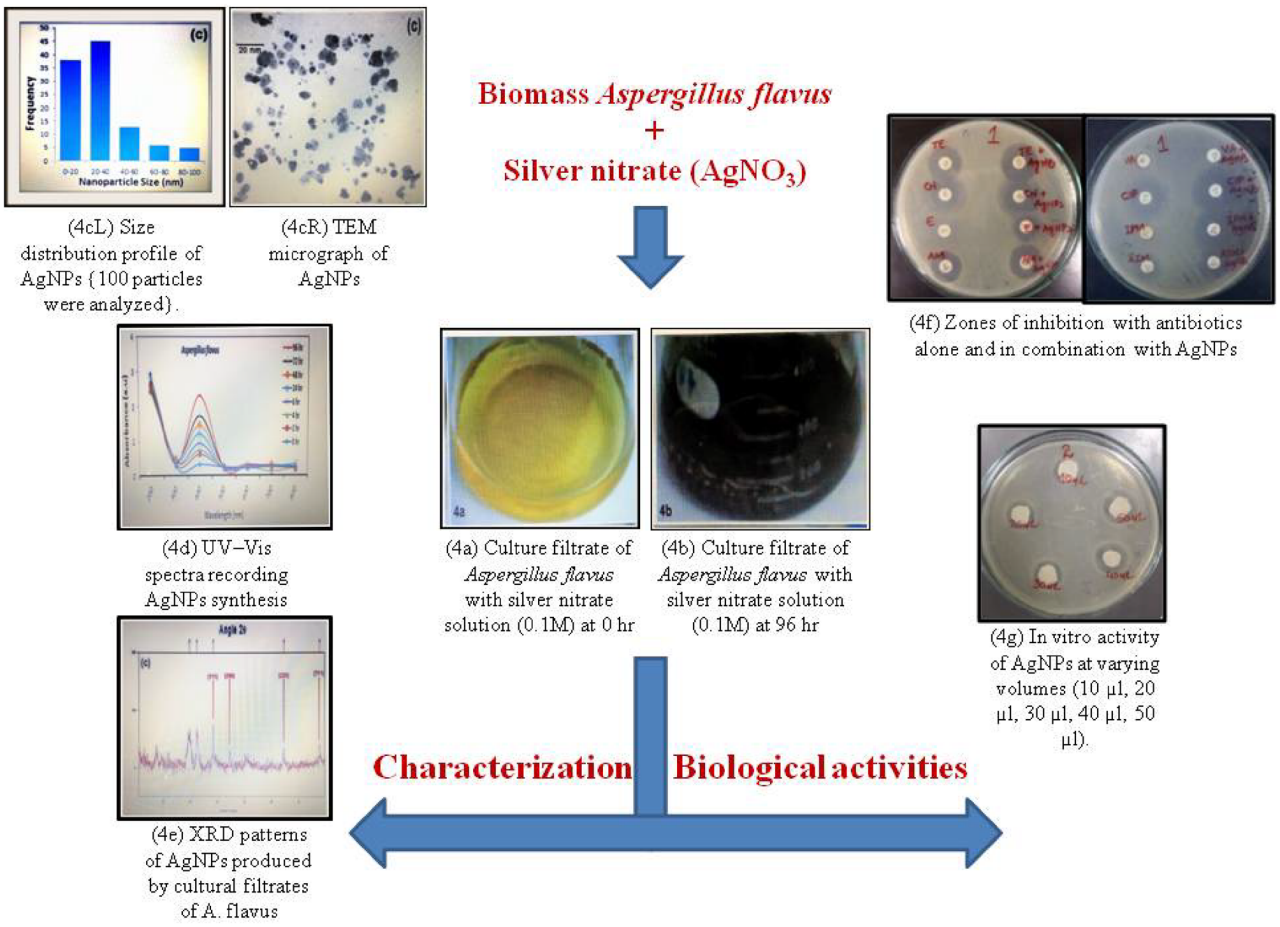
Biosynthesis of AgNPs from *Aspergillus flavus* having antibacterial activity. (4a) Culture filtrate of *A. flavus* with silver nitrate solution (0.1 M) at 0 h. (4b) Culture filtrate of *A. flavus* with silver nitrate solution (0.1 M) at 96 h. (4cL) Size distribution profile of AgNPs synthesized by cultural filtrates of *A.* *flavus* {100 particles were analyzed}. (4cR) TEM micrographs of AgNPs produced by cultural filtrate of *A. flavus.* (4d) UV–Vis spectra recorded for the biosynthesis of AgNPs in response to 0.1 M. silver nitrate inoculated media of *A. flavus.* (4e) XRD patterns of AgNPs biosynthesized by cultural filtrates of *A. flavus.* (4f) Zones of inhibition with antibiotics alone and in combination with AgNPs*.Antibiotics in combination with AgNPs (A and B) have a wider zone of inhibition as compared to antibiotic alone showing thereby that AgNPs conjugated to antibiotics have a stronger antibacterial potency. (4 g) In vitro activity of AgNPs* at varying volumes (10 µL, 20 µL, 30 µL, 40 µL, 50 µL). *AgNPs* silver nanoparticles.

A volume of 200 µL of 0.5 McFarland bacterial suspension was prepared and added to LB broth from the 1st to the 10th well. Serial dilutions from stock solutions of AgNPs (1 mg/10 mL) were added to 96-well round-bottom microtiter plates (Thermo Fischer Scientific, UK) to finalize a 2 µg/mL concentration. Petri plates were incubated overnight at 37 °C following which the plates were observed to determine the absence of growth comparing each well with positive and negative controls^40^. MBC (minimum bactericidal concentration) measures the first dilution showing no growth on the agar. Wells showing no visible growth in the microtiter plate were further inoculated on nutrient agar (Oxoid, UK). A schematic illustration is shown (Fig. 1).

### Statistical analysis

Statistical analysis was performed for evaluating the percentage of resistance in relation to the ward and type of sample. Association between the type of sample [urine, sputum, wound, blood, pus, and tissue] and antibiotic resistance gene were evaluated by Chi-Square test. A p-value ≤ 0.05 was considered statistically significant. As per Pearson-correlation coefficient (r), the association between the phenotypic and genotypic variables was determined as follows: weak correlation: |r|< 0.3, moderate correlation: 0.3 <|r|< 0.5, strong correlation: 0.5 <|r|< 0.85^41^. Statistical analyses were carried out using SPSS software version 20 (IBM Corp., Armonk, NY, USA).

## Results

### Phenotypic characteristics of the recovered *Pseudomonas aeruginosa* isolates

*Pseudomonas aeruginosa* counts as a heterotrophic and motile, Gram-negative bacterium that is rod-shaped measuring 1–5 µm and 0.5–1.0 µm. It has been documented as a facultative aerobe that has the ability to grow through both aerobic and anaerobic respiration with nitrate studied to be the final electron acceptor. In accordance with Magiorakos *et al*., the tested isolates would fall in the category of MDR since the isolates were resistant to a minimum of one antimicrobial drug in three or exceeding antibiotic categories^31^. *Pseudomonas aeruginosa* has the ability to produce elastase and protease enzymes as well as hemolysins.

### Antibiotic susceptibility testing

**Figure 2:**
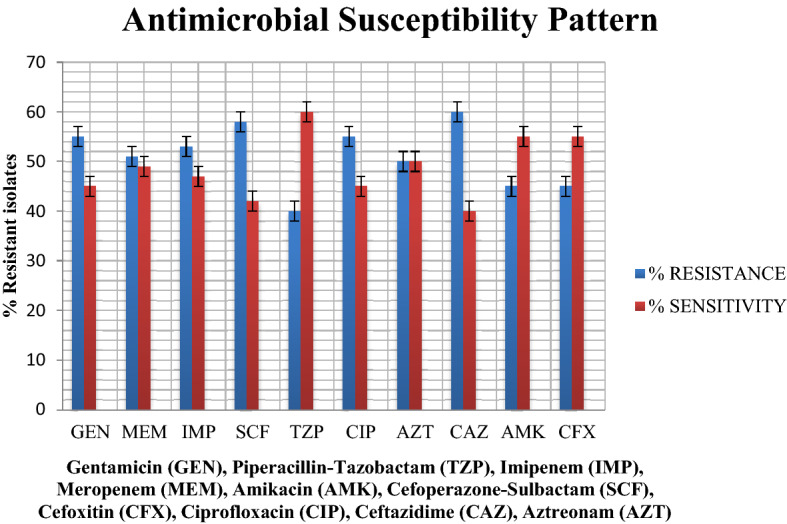
Percentage antibiotic resistance and sensitivity *Pseudomonas aeruginosa* isolated from patients in Punjab, Pakistan (n = 255).

Sensitivity testing was accomplished on MH agar (Oxoid) as per recommendations of CLSI 2019 (Clinical and Laboratory Standards Institute 2019)^20^. This has been illustrated in Fig. 2. The pattern of antimicrobial resistance in *Pseudomonas aeruginosa* in association with departments has been tabulated in Table 3.

**Table 3:** Pattern of antimicrobial resistance in *Pseudomonas aeruginosa* in association with departments.

Antibiotic	Wards							
	Surgery	Medicine	Orthopaeds	ICU	ENT	Gynaecology	Total	p-value*
Meropenem	56	42	4	14	8	6	130 (51%)	0.000
Imipenem	62	27	21	9	7	9	135 (53%)	0.005
Gentamicin	51	44	25	10	4	6	140 (55%)	0.001
Cefoxitin	43	30	10	24	2	5	114 (45%)	0.000
Ciprofloxacin	51	51	22	8	4	4	140 (55%)	0.000
Ceftazidime	65	35	16	14	10	13	153 (60%)	0.062
Aztreonam	39	32	28	18	5	6	128 (50%)	0.001
Pip-Tazo	35	25	19	16	3	4	102 (40%)	0.048
Amikacin	51	35	5	9	5	10	115 (45%)	0.001
Cefoperazone-Sulbactam	76	39	11	10	5	7	148 (58%)	0.000

Vertical ranking enlists antibiotics. Horizontal ranking enlists the department of sample collection.

*p value ≤ 0.05 was considered statistically significant.

### Phenotype detection of carbapenemase activity

**Figure 3:**
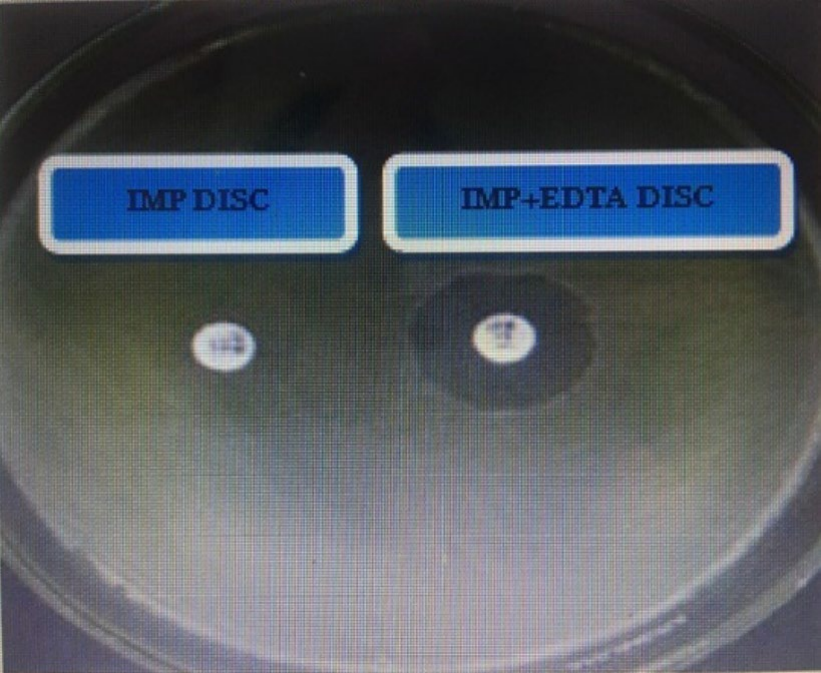
CDST^a^ for detection of MBL producers of *Pseudomonas aeruginosa* isolated from Punjab, Pakistan. This test shows using a disc of IMP^b^ alone and IMP/EDTA disc as per me﻿thodology of Wadekar *et al*.^24^. A wider zone of inhibition is measured with IMP/EDTA disc as compared to IMP disc alone. This identifies metallo β-lactamase producers. ^a^*CDST* Combined disc synergy disc; ^b^Imipenem.

**Figure 4:**
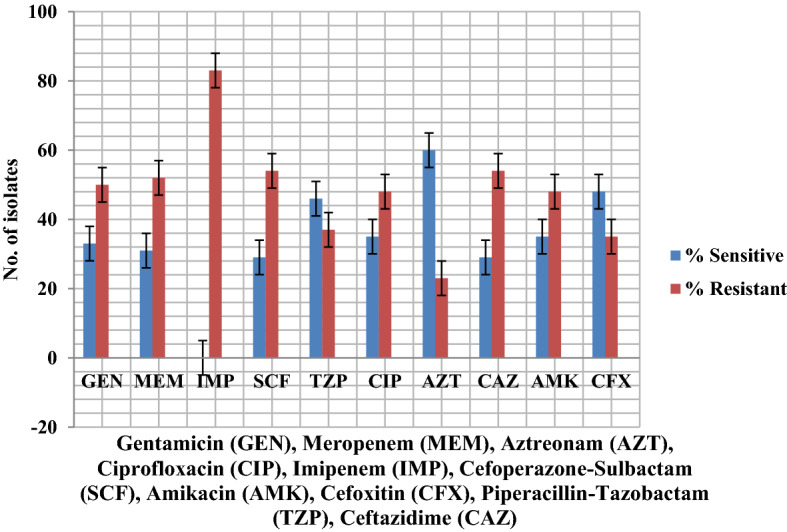
Antibiotic susceptibility testing of MBL producing *Pseudomonas aeruginosa* (n = 83). *MBL* metallo β-lactamase.

Of 255 total isolates, 61.5% were carbapenemase producers, including 32 samples from urine (45%), wound swabs n = 25 (28%), sputum n = 28 (80%), blood n = 21 (70%), tissue n = 5 (41%) and pus n = 4 (22%). Phenotypic detection of Metallo-β-lactamases was observed by CDST as per guidelines of CLSI 2019 (Fig. 3). Antibiotic susceptibility of MBL producers is illustrated in Fig. 4. Prevalence of MBL and ESBL producers in correlation with the department is shown in Table 4.

**Table 4:** Prevalence of MBL- and ESBL-producing *Pseudomonas aeruginosa* in correlation with department.

Isolates	Surgery	Medicine	Ortho	ICU	ENT	Gynaecology	Total	p-value*
*bla*_IMP_ (n = 10)	7 (70%)	2 (20%)	0 (0%)	0 (0%)	0 (0%)	1 (10%)	10 (100%)	0.26
*bla*_VIM_ (n = 12)	9 (75%)	2 (16.7%)	0 (0%)	0 (0%)	0 (0%)	1 (8.3%)	12 (100%)	0.101
*bla*_SHV_ (n = 25)	19 (76%)	3 (12%)	0 (0%)	1 (4%)	1 (4%)	1 (4%)	25 (100%)	0.002
*bla*_TEM_ (n = 34)	18 (52.9%)	13 (38.2%)	0 (0%)	2 (5.9%)	1 (2.9%)	0 (0%)	34 (100%)	0.012
*bla*_OXA_ (n = 17)	8 (47.1%)	7 (41.1%)	0 (0%)	0 (0%)	2 (11.7%)	0 (0%)	17 (100%)	0.099
*bla*_AmpC_ (n = 17)	12 (70.5%)	1 (5.9%)	3 (17.6%)	1 (5.9%)	0 (0%)	0 (0%)	17 (100%)	0.043
Total	73	28	3	4	4	3	115	

Vertical ranking shows antibiotic resistance genes. Horizontal ranking shows department of sample collection.

*p value ≤ 0.05 was considered statistically significant.

### Multiplex PCR for AMR genes (*bla*_OXA_, *bla*_IMP_, *bla*_TEM_, *bla*_SHV_, *bla*_VIM_)

**Figure 5:**
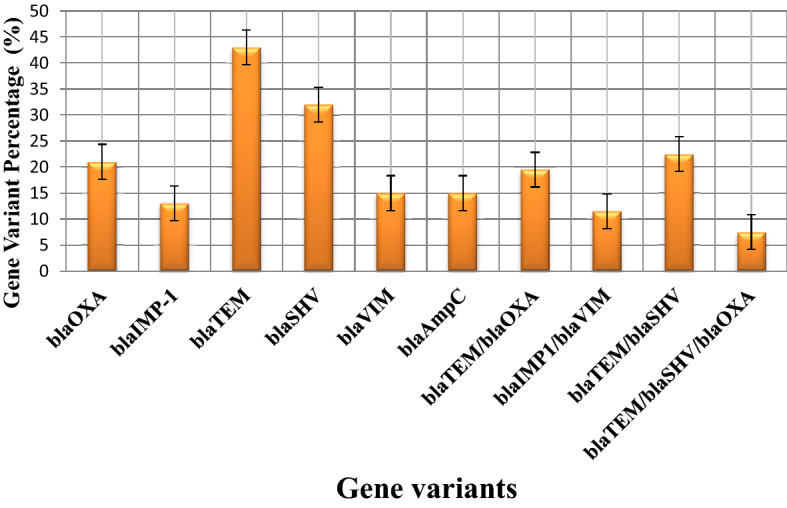
Co-expression of gene variants (MBL and ESBL) in isolates of *Pseudomonas aeruginosa.*

Multiplex PCR detected the existence of resistance genes in 52.5% (n = 80) of the ESBL producers. Expression of *bla*_TEM_ was of the order 43% of ESBL producers (n = 34) whereby *bla*_SHV_ was detected in 32% of isolates. Likewise, the expression of *bla*_OXA_ was 21%. *bla*_VIM_ along with *bla*_IMP-1_ co-existed in 11.5% of MBL producers. 57.5% ESBL-positive strains exhibited simultaneous existence of *bla*_TEM_, *bla*_SHV_ plus *bla*_OXA_, *bla*_TEM_ was concomitantly expressed with *bla*_OXA_ types in 19.5% of ESBL producers whereas *bla*_TEM_ simultaneously showed presence with *bla*_SHV_ in 22.5% isolates. Co-existence of *bla*_OXA_ plus *bla*_SHV_ was found in 9.5% of isolates while *bla*_SHV_, *bla*_TEM_, as well as *bla*_OXA_ subsisted in 7.5% of ESBL-producers. Expression of *bla*_AmpC_ was positive in 15% of isolates that were cefoxitin resistant. The correlation between phenotypic and genotypic MDR has been tabulated in Table 5. The co-expression of various genes is depicted in Fig. 5.

**Table 5:** Correlation matrix of phenotypic and genotypic MDR isolates of *Pseudomonas aeruginosa* by Pearson correlation co-efficient.

	MBL producer	*bla*_IMP_	*bla*_VIM_	*bla*_SHV_	*bla*_TEM_	*bla*_OXA_	*bla*_AmpC_	ESBL producer	
MBL Producer	X	0.291 0.000	0.320 0.000	− 0.06 0.339	− 0.149 0.017	− 0.085 0.176	− 0.018 0.776	0.710 0.256	Statistics {p =}*
*bla*_IMP_	0.291 0.000	X	0.909 0.000	0.001 0.983	0.04 0.529	0.027 0.668	0.027 0.668	0.038 0.550	
*bla*_VIM_	0.320 0.000	0.909 0.000	X	− 0.011 0.861	0.022 0.729	0.015 0.813	0.015 0.813	0.009 0.881	
*bla*_SHV_	− 0.06 0.339	0.001 0.983	− 0.011 0.861	X	0.569 0.000	0.282 0.000	0.229 0.000	0.488 0.000	
*bla*_TEM_	− 0.149 0.017	0.04 0.529	0.022 0.729	0.569 0.000	X	0.589 0.000	0.126 0.044	0.555 0.000	
*bla*_OXA_	− 0.085 0.176	0.027 0.668	0.015 0.813	0.282 0.000	0.589 0.000	X	− 0.008 0.894	0.395 0.000	
*bla*_AmpC_	− 0.018 0.776	0.027 0.668	0.015 0.813	0.229 0.000	0.126 0.044	− 0.008 0.894	X	0.294 0.000	
ESBL Producer	0.071 0.256	0.038 0.550	0.009 0.881	0.488 0.000	0.555 0.000	0.395 0.000	0.294 0.000	**X**	
Pearson correlation coefficient (r =)**									

*p value ≤ 0.05 was considered statistically significant.

**Based on Pearson-correlation coefficient (r), the association between the phenotypic and genotypic variables was determined as: |r|< 0.3; weak correlation, 0.3 <|r|< 0.5; moderate correlation, 0.5 <|r|< 0.85; strong correlation.

### MIC of MBL producing *Pseudomonas aeruginosa*

The MIC of imipenem against 10 (13%) *bla*_IMP_-producing *Pseudomonas aeruginosa* isolates inhibited five isolates at 16 μg/mL and five other isolates at 8 μg/mL. The MIC of imipenem for 12 (15%) *bla*_VIM_-producing *Pseudomonas aeruginosa* was 16 μg/mL, for three isolates was 8 μg/mL, and for the remaining three isolates measured 32 μg/mL. MIC of Imipenem for 17 (21%) *bla*_OXA_ producers was 16 μg/mL for 12 isolates plus 8 μg/mL for five isolates.

### Characteristics of AgNPs

#### Visual observations

Changes in the color of the reaction mixture were observed and images were recorded as the initial and ending stages of the experiment (Figs. 1–4a,b).

#### UV-spectroscopy

**Figure 6:**
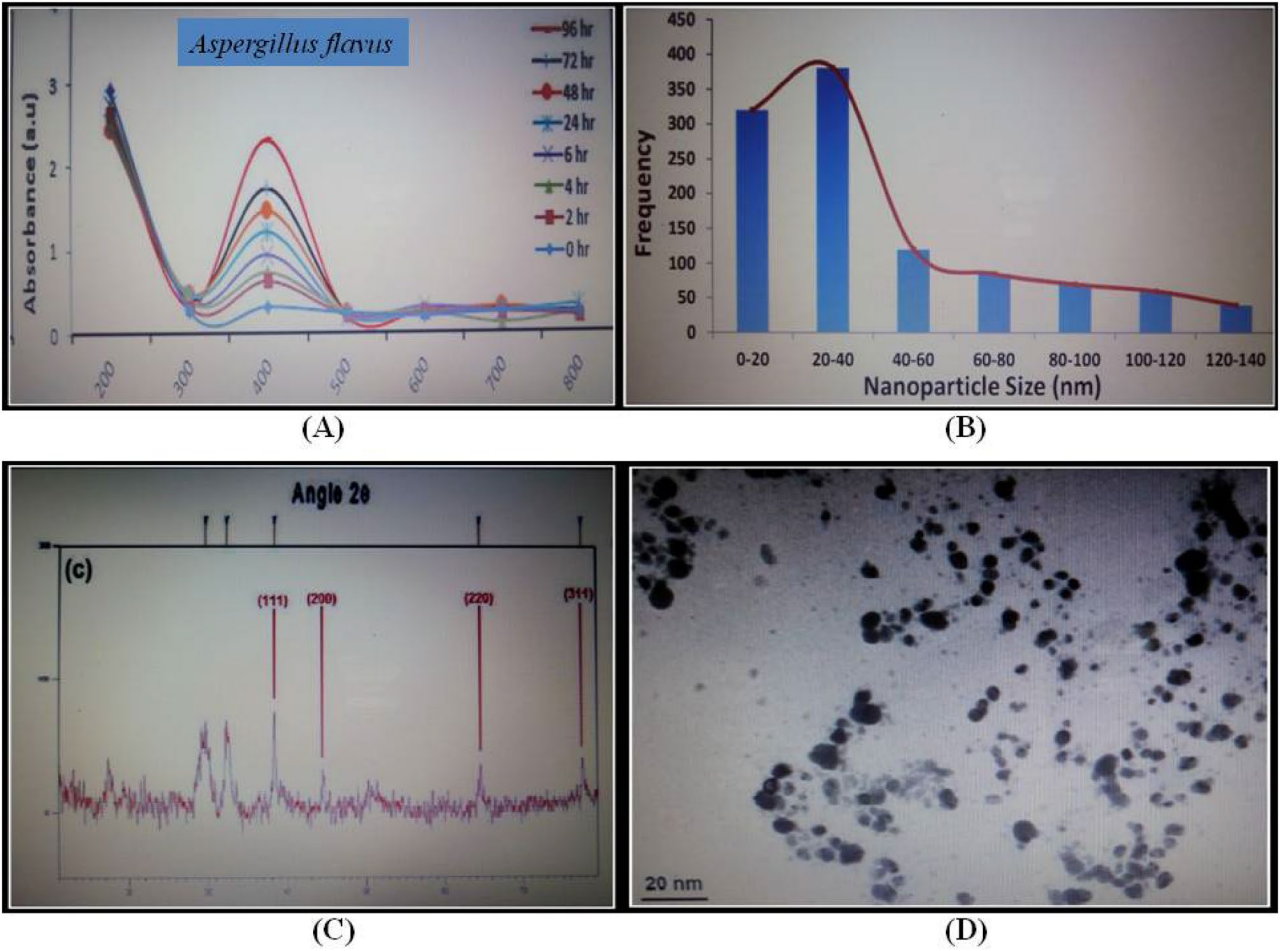
Characterization of AgNPs biosynthesized using *Aspergillus flavus.* (**A**) UV–Vis spectra recorded for the biosynthesis of AgNPs in response to 0.1 M silver nitrate inoculated media of *A. flavus.* (**B**) Size distribution profile of AgNPs synthesized prepared by cultural filtrates of *A. flavus* {A total number of 100 particles were analyzed}. (**C**) XRD patterns of AgNPs biosynthesized from cultural filtrates of *A. flavus.* (**D**) TEM micrographs of AgNPs produced by cultural filtrates of *A. flavus.*

At the time of incubation, the reaction mixture’s UV–visible spectroscopy recorded spectra of increased intensity having a range of 350–600 nm; crucial peaks occurred at approximately 400–470 nm (Fig﻿. 6). Moderately increasing peak absorbance accompanied with time was most notably connected with a change in the reaction mixture’s color exhibiting AgNPs synthesis positively (Fig. 6A). Particle size histogram has been depicted (Fig. 6B).

#### XRD analysis

The crystalline nature of the AgNPs was depicted using the Debye–Scherrer analysis along with XRD of the dried sample’s drop-coated film. XRD analysis revealed four vital peaks in the total spectrum of 2θ value that extended in the range of 20°–80°. The crystallites averaged in size from 13 to 26 nm. Debye–Scherrer formulae were used to analyze the silver nanocrystallites ranging from 21 nm from total breadth at peak’s half maximum. The Debye–Scherrer equation is derived from Bragg’s law that determines the diameter of crystal samples according to the formula given below;

$${\text{D }} = k.\lambda ,$$

$$\beta \cos \theta ,$$

where D is the mean diameter, λ is the wavelength, k is the shape factor (0.9), θ is the Bragg angle for studied diffraction, β is the the full width at half maximum. XRD diractograms demonstrated four vital peaks around 2θ angles at 38, 44, 64, and 77. Crystallites estimated via XRD technique revealed size in the nanometer range (Fig. 6C).

#### TEM of AgNPs

Bright-field image mode was employed for the analysis of samples. Ultra-sonication produced pure ethanol based dilute suspensions of AgNPs. The suspension was spread drop-wise on 300-mesh lacy copper grids that were coated with carbon followed by drying for scanning under JEOL-1010 TEM. Accelerating high voltage was kept at 80 kV. Required adjustments and alignments were done following the selection of apertures and sample images were scanned on screen. Micrographs were recorded at definite magnifications on the photographic plates by focusing on the sample grids at the correct places. Standard developing procedures were adopted for processing the exposed photographic plates. These were scanned (flatbed high-resolution scanner) to finally achieve the image positives. TEM micrographs showed nanoparticles of different shapes where predominantly the shape was spherical. AgNPs acquired a size range of 5–30 nm^30,34^. The majority of the AgNPs were spread out in the micrographs with only a few places revealing larger aggregates of differing sizes (Fig. 6D).

#### Scanning electron microscopy (SEM)

**Figure 7:**
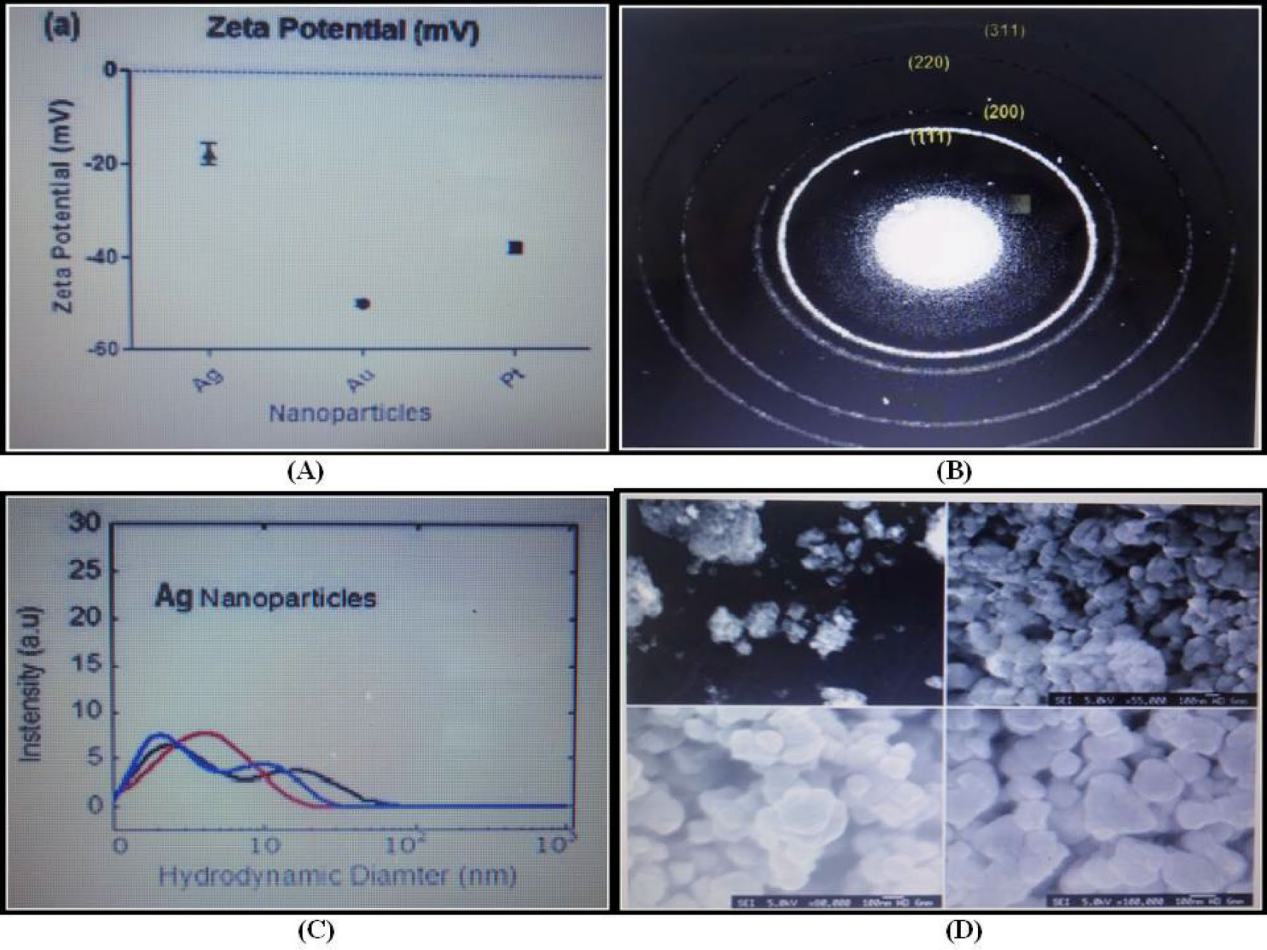
Myco-genized AgNPs (**A**) Zeta potential measurements for mycogenized AgNPs. (**B**) SAED analysis (**C**) Dynamic Light Scattering (DLS) measurements (**D**) SEM micrographs of AgNPs.

Scanning electron micrographs were acquired with the JEOL 5600 following filtration of the samples through Millipore filters of 0.2 µm pore size to remove any contaminants that could possibly interfere with the SEM images. Samples for analysis were prepared by overnight fixation with 2.5% glutaraldehyde at room temperature. Subsequently, dehydration of the sample was carried out with gradient alcohol (10–95%) followed by incubation for 20 min in every gradient and soaked in absolute alcohol for about 2–5 min. Approximately 25 µL of the sample was pipetted out and loaded onto a ‘stub’ supplied for SEM analysis. The stub is approximately 1 cm in diameter has a cylindrical shape and is made of copper. Scanning of SEM was performed at the accelerating voltage of 25 kV from a distance of 5 mm to 5 cm (Fig. 7D).

#### Scattered area electron diffraction (SAED)

The crystalline character of NPs was determined by employing the SAED analysis supplementarily with TEM. Diffractions were specifically acquired at a distance of 80 cm. Myco-genized AgNPs were majorly crystalline in nature, which could be perfectly listed to the Bragg reflections of the face-centered cubic (fcc) nature of the crystalline silver (Fig. 7B).

#### Nano-suspensions of these myco-genized metallic NPs

Approximately 100 ppm of NP was diffused in test tubes that contained 100 mL of sterile deionized water to obtain nano-suspensions.

#### Dynamic light scattering (DLS)

Determination of the AgNPs size distribution was done by dynamic light scattering measurements on Malvern Zeta Sizer Nano ZS (Malvern Instruments Ltd., UK) using disposable clear zeta cells (DTS 1060C). The instrument allowed for an average diameter along with polydispersity index (PDI). Recordings for zeta average diameter plus PDI reported herewith were obtained by calculating an average of three separate measurements where each measurement was recorded after ten repetitions on each sample (Fig. 7C).

#### Zeta potential measurement

Zeta potential analysis is for the determination of the surface charge of AgNPs in solution form. The magnitude of zeta potential predicts the stability of the colloids. AgNPs having Zeta potential value exceeding + 25 mV or lesser than a value of − 25 mV possess a higher probability of stability. Dispersions having lower zeta potential value clump because of inter-particle Van Der Waal attractions.

Zeta potential measurements were executed with the AgNPs obtained from stock solution along with the resuspended employing Malvern Instruments Zeta-sizer Nano (Malvern Instruments Ltd., UK) that operated with a variable power of (5–50 mW) together with He–Ne laser at 632 nm. Measurements were obtained in zeta cells (DTS 1060C) at a temperature of 25 °C that were independently recorded thrice. Flow through the cell underwent washing three times before and during measurements with ultrahigh pure water prior to the addition of the subsequent sample. AgNPs zeta potential was evaluated at pH 7.4. Zeta potential measurements for AgNPs revealed that the maximum value was around − 19 mV (Fig. 7A).

### MIC and MBC of AgNPs (µg/mL)

**Figure 8:**
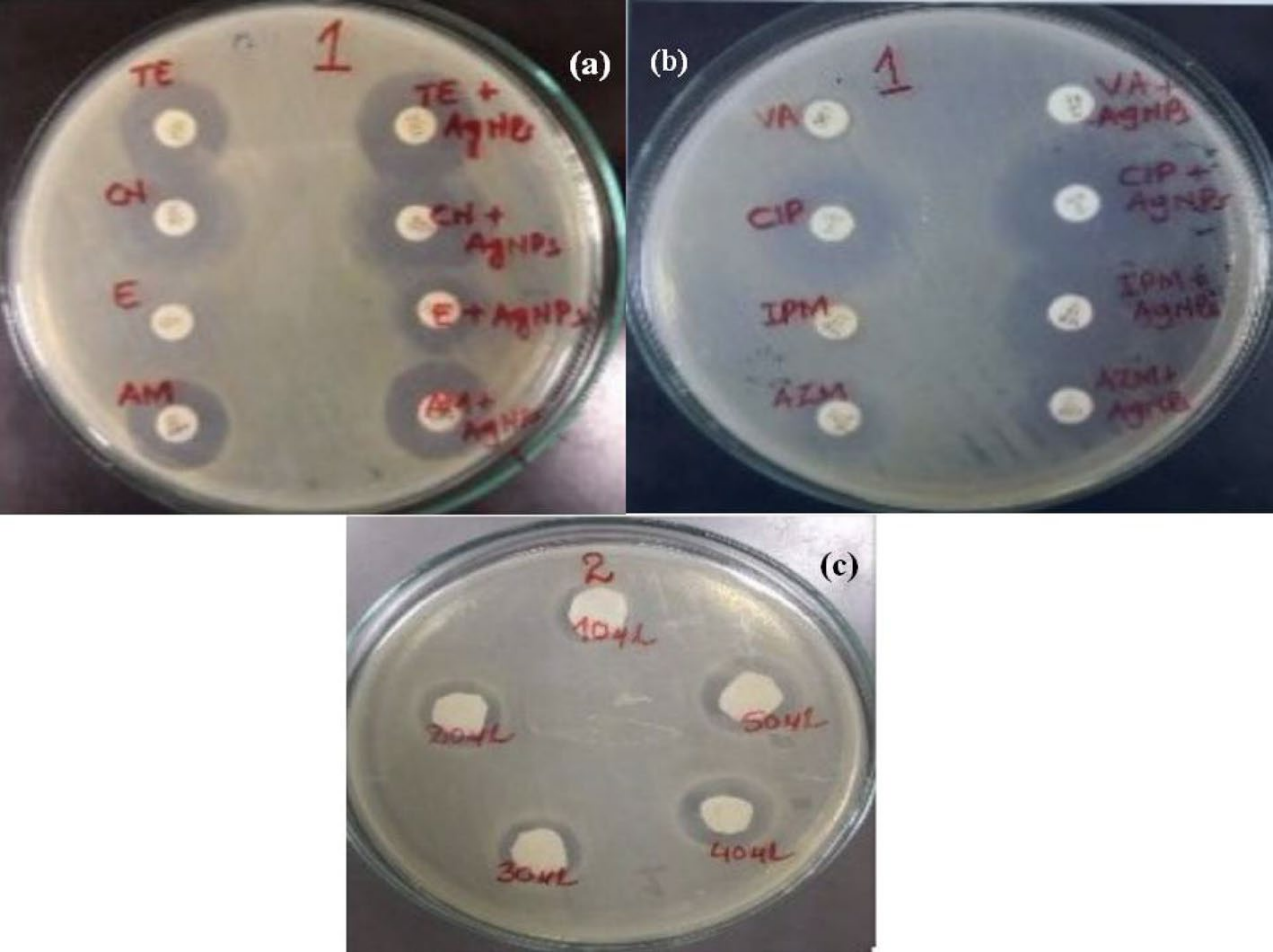
Antimicrobial effects: (**a**,**b**) AgNPs and antibiotic-AgNPs combinations, (**c**) AgNPs activity at varying volumes.

The MIC value for AgNPs was 1 µg/mL, and MBC was 2 µg/mL against MBL-producing *Pseudomonas aeruginosa*. The results depict that AgNPs conjugated with antibiotics are effective in the case of multidrug-resistant *Pseudomonas aeruginosa* and can be employed as an alternate treatment option following various clinical studies. The antibacterial potency of AgNPs was analyzed by recording the diameter of zones of inhibition in millimetres. This is illustrated in Fig. 8a,b.

### Effect of AgNP solution volume on antibacterial action

Different volumes (10–50 μL) of 100 ppm AgNP solution were used to assess the impact of suspension volume on inhibition zone size. In the case of each test isolate, an increase in the inhibition zone was monitored with the corresponding rise in AgNP solution volume as shown in Fig. 8c.

### Synergistic antibacterial effect of antibiotics in conjugation with AgNPs

The combined effect of AgNPs in conjugation with multiple antibiotics was construed against multidrug-resistant *Pseudomonas aeruginosa* using the disk diffusion method. It was observed that the antibacterial potency of the antibiotics was upgraded in the presence of AgNPs. The reducing order of antimicrobial activity of antibiotics and AgNPs plus their combinations was piperacillin/tazobactam + AgNPs (31 ± 1.4 mm), cefoxitin + AgNPs (30 ± 1.0 mm) > amikacin + AgNPs (25 ± 1.3 mm) > aztreonam + AgNPs (23 ± 1.5 mm) > meropenem + AgNPs (22 ± 1 mm) > imipenem + AgNPs (20 ± 1.5 mm) > gentamicin + AgNPs (17 ± 0.5 mm) > ciprofloxacin + AgNPs (16 ± 0.1 mm) > cefoperazone/sulbactam + AgNPs (14 ± 0.4 mm) ≥ ceftazidime + AgNPs (14 ± 1.2 mm). The combined effect of AgNPs plus antibiotics showed a 0.15–3.51 (average, 2.09) fold-area augmentation of the antibacterial activity. The potency of AgNPs alone and in combination with antibiotics was ascertained by measuring the inhibition zones. The inhibition zones were measured in a range of 14–31 mm. AgNPs used alone led to weaker bactericidal activities. Combinations of standard antibiotics with AgNPs ensued in a 0.15–3.51 (average 2.09) fold-area augmentation of the antibacterial activity. Typically, the highest zone of inhibition(s) was recorded by piperacillin/tazobactam in combination with AgNPs. Isolates resistant to ceftazidime showed a zone of inhibition measuring 14 ± 1.2 mm in combination with AgNPs (Table 6).

**Table 6:** Single and combined efficacy of AgNPs and antibiotics against MDR *Pseudomonas aeruginosa.*

ZOI (mm) of antibiotics in combination with AgNPs	*Pseudomonas aeruginosa*
**Piperacillin/tazobactam (A) (100 µg)**	18 ± 0.5
AgNP	13 ± 0.6
Piperacillin/tazobactam + AgNP (B)	31 ± 1.4
Fold increase of inhibition zone area^b^	1.97 (72.2%)
**Cefoxitin (A) (30 µg)**	18 ± 0.3
AgNP	13 ± 0.5
Cefoxitin + AgNP (B)	30 ± 1.0
Fold increase of inhibition zone area^b^	1.8 (66.6%)
**Amikacin (A) (30 µg)**	16 ± 0.7
AgNP	13 ± 0.6
Amikacin + AgNP (B)	25 ± 1.3
Fold increase of inhibition zone area^b^	1.4 (56.2%)
**Aztreonam (A) (10 µg)**	14 ± 0.4
AgNP	13 ± 0.2
Aztreonam + AgNP (B)	23 ± 1.5
Fold increase of inhibition zone area^b^	1.7 (64.2%)
**Meropenem (A) (10 µg)**	12 ± 1
AgNP	13 ± 0.5
Meropenem + AgNP (B)	22 ± 1
Fold increase of inhibition zone area^b^	2.3 (83.3%)
Imipenem (A) (10 µg)	10 ± 1.5
AgNP	13 ± 0.5
Imipenem + AgNP (B)	20 ± 1.5
Fold increase of inhibition zone area^b^	3.0 (100%)
**Gentamicin (A) (10 µg)**	8 ± 0.1
AgNP	13 ± 0.4
Gentamicin + AgNP (B)	17 ± 0.5
Fold increase of inhibition zone area^b^	3.5 (112.5%)
**Ciprofloxacin (A) (5 µg)**	8 ± 1.2
AgNP	13 ± 0.6
Ciprofloxacin + AgNP (B)	16 ± 0.1
Fold increase of inhibition zone area^b^	3.0 (100%)
**Cefoperazone/sulbactam (A) (75–10 µg)**	8 ± 0.5
AgNP	13 ± 1.3
Cefoperazone/sulbactam + AgNP (B)	14 ± 0.4
Fold increase of inhibition zone area^b^	2.1 (75%)
**Ceftazidime (A) (30 µg)**	0^a^
AgNP	13 ± 0.3
Ceftazidime + AgNP (B)	14 ± 1.2
Fold increase of inhibition zone area^b^	0.15 (133.3%)

Rank order is indicated by Alphabets. Vertically, the ranking is linked to combinations of antibiotics against bacterial isolates; horizontally, the ranking is of bacterial isolates with respect to sensitivity to antibiotic amalgamation (P, 0.05). ^**a**^Disc diameter (6 mm) was employed for calculation of fold increase in absence of bacterial growth inhibition zone^34^. ^b^Fold increase of inhibition zone area = (B^2^ − A^2^)/A^2^

$${\text{As }}\% {\text{ synergism}} = \, \left( {{\text{B}} - {\text{A}}} \right)/{\text{A}} \times {1}00$$^.^

*AgNP* silver nanoparticles.

## Discussion

The ability to produce Metallo-β-lactamases is the most vital mechanism by which *Pseudomonas aeruginosa* gains antimicrobial resistance against several drugs. Rapidly emerging strains of *Pseudomonas aeruginosa* that can produce MBLs are an urgent concern for hospitals and healthcare centers^42^. Hospital environment in lower middle economic countries has certain factors that contribute to the spreading of infections by MDR bacteria. These are improper handling of medical instruments, contaminated hands of medical personnel, and various unhygienic surfaces like floors and doorknobs^42^.

Multidrug-resistant phenotypes in *Pseudomonas aeruginosa* result from different mechanisms that are found to interact with one another thus providing antimicrobial resistance^43^. Increased expression of the efflux pump, reduction in the porins present in the external membrane, and modified geometry of the penicillin-binding proteins contribute to acquired resistance^43^. Acquisition of dual resistance to more than a single class of antimicrobials calls for reasoned treatment of infections owing to *Pseudomonas aeruginosa*^39,43^. Isolates of *Pseudomonas aeruginosa* resistant to ceftazidime and meropenem exhibit MBL and ESBL activity confirming that MBL-producing genes are considered crucial to resistance against these antimicrobials^25,29^.

In this study, more isolates were obtained from samples collected from female patients (145/523) as compared to male patients (110/636). These findings differ from a study that was carried out in Germany whereby out of a total of 168 patients, 67.3% of males, and 32.7% of females tested positive^44^. The highest isolates of *Pseudomonas aeruginosa* were from patients aged 40–49 years (25.4%). These results differ from another study that reported patients aged more than 55 years having a higher prevalence of *Pseudomonas aeruginosa*^45^. This study shows a much-increased prevalence of infections caused by *Pseudomonas aeruginosa* owing to poor health and sanitary condition of patients in hospital environments, improper prescription of antimicrobial drugs, and the presence of antimicrobial resistance in bacteria^46^. Almost 54.5% of the MDR strains of *Pseudomonas aeruginosa* were observed by Saderi *et al*.^47^. This increase in resistance against antibiotics may be due to activated efflux pumps in bacteria, modification of the target site of drugs, the presence of reducing enzymes, or the loss of membrane proteins^48^. Strains of *Pseudomonas aeruginosa* isolated in this study showed higher resistance against carbapenem drugs, i.e., imipenem (53%) and meropenem (51%). These findings are synonymous with a study conducted in the United States that mentions 65% resistance to carbapenem drugs^49^. The MIC values of meropenem and imipenem were in the range of 8 μg/mL to ≥ 32 μg/mL. These results agree with a previous study whereby the MIC of imipenem was > 32 μg/mL^50^. Results of the current study have intimated a prevalence of MBL producers as 61.5% of which 11.5% expressed *bla*_IMP-1_/*bla*_VIM_ while *bla*_TEM_/*bla*_OXA_ were expressed in 19.5% and *bla*_OXA_/*bla*_SHV_ were concomitantly expressed in 9.5%. Tahmasebi *et al*. have reported the expression of 12 phenotypically MBL-producing isolates of *Pseudomonas aeruginosa*, of which PCR amplification confirmed *bla*_VIM_ in 33.3%, and *bla*_IMP_ in 25% of isolates^41^.

The antibacterial activities of silver have been discussed globally. Recently, thiol-dependent enzymes including thioredoxin (Trx) plus glutathione (GSH) systems have been discovered as potential bactericidal targets in MDR bacteria^16,51^. Reports have concluded that silver acts in conjugation with ebselen inhibiting the Trx system resulting in quick depletion of GSH in Gram-negative bacteria^16,51^. Additionally, silver augments the bacterial sensitivity towards antibiotics by blocking the Trx system^16^. Concomitantly, reactive oxygen species (ROS) generate helping bacteria become sensitive to conventional antibiotics^16,51^. Furthermore, the antibiotic- AgNPs combination strongly binds to the bacterial cells promoting the release of Ag(+) and resulting in an increased concentration of Ag(+) in the vicinity of bacteria. These findings are in support of the theory that Ag(+) release from AgNPs is the potential agent that causes toxicity^52,53^.

Minuscule AgNPs as found in the current study having a spherical shape possessing a microscale diameter are more sensitive to release silver because of enhanced surface area. Furthermore, AgNPs have the ability to penetrate the cell walls of bacteria by modifying their structure due to their nanoscale size^14,52^. Disruption of the cellular membranes can lead to rupture of the organelles resulting in cellular lysis^13,14,39^. The smaller size of nanoparticles in this study (5–30 nm) is attributed to their high efficiency in penetrating bacterial cells as compared to previous studies where the size of nanoparticles was 65–90 nm in diameter^14^. The collaborative effect of AgNPs plus antibiotics showed a 0.15–3.51 (average, 2.09) fold-area augmentation of the antibacterial activity. Several studies have recognized the antimicrobial potency of silver nanoparticles in combating bacteria. Unfortunately, the precise mechanism involved has not yet been ascertained^44^.

AgNPs can be toxic due to the release of ionic Ag in combination with surface properties, shape, and size. Lesser toxicity is associated with prismatic and cubic geometries of AgNPs at a concentration of ˂ 100 µg/L^54^. Auclair *et al*. have determined that sublethal toxicity ascertained at 96 h on the basis of salient characteristic morphological changes exhibits the following toxicity: ionic (2.6 µg/L), spherical (22 µg/L), and prismatic (32.5 µg/L) AgNPs^54^. Auclair *et al*. have also concluded that the structure of nanocube was not toxic at this concentration while nanoparticles possessing a low aspect ratio combined with high circularity as well as elongation properties exhibit high toxicity at both sublethal and lethal levels. AgNPs shape has been studied to influence the toxicity demanding further research in the field for understanding the mechanisms playing part in making AgNPs toxic^54^. Furthermore, varying neurobehavioral effects have been reported by Vogt *et al*. for various coatings and sizes of AgNPs while studying exposure of larvae to Ag + which suggests that AgNPs potentially act as a neurobehavioral disruptor^55^. *Fu et al﻿*. have reported that exposure to AgNPs impairs social behavior and learning in the subjects studied which indicates a strong neurotoxic effect^56^. Bhalodia *et al*. reported an MIC ranging from 1.406 to 5.625 µg/mL plus an MBC ranging from 2.813 to 5.625 µg/mL for Metallo-β-lactamase producers in *Pseudomonas aeruginosa*^35^.

Uncoated AgNPs promote significant cytotoxic effects on PBMCs at proportionately lesser concentrations (< 5 μg/mL) and shorter exposure times (3–12 h). PBMCs are cells of the immune system that constitute lymphocytes (T cells, B cells, and NK cells) plus monocytes. This suggests that the coating of AgNPs decreases the interacting active surface area sites with the cellular components^57,58^. It has been reported by Kim *et al*. that AgNPs exhibit genotoxic effects in BEAS-2B cells that are bronchial epithelial cell lines^59^. The oxidative stress promoted by AgNPs might be a pivotal element in the genotoxic effects caused by AgNPs^59^. AgNPs have been studied to cause damage in DNA while creating formation of the micronucleus in a dose-dependent approach. The specific AgNPs dose-dependent activity due to the formation of reactive oxygen radicals has been studied to be reduced by superoxide dismutase most importantly as shown in the cytokinesis-block MN assay as well as the comet assay. The present study demonstrates that AgNPs conjugated with antibiotics inhibited *Pseudomonas aeruginosa* as the zone of inhibition significantly increased with the use of combined discs.

## Conclusions

The current study concludes the high prevalence of multidrug-resistant *Pseudomonas aeruginosa* whereby a positive correlation has been observed between phenotypic and genotypic variants of *Pseudomonas aeruginosa*. Nevertheless, the antibiotic-silver nanoparticles combination has shown antibacterial potency against MDR *Pseudomonas aeruginosa*. Combinations of AgNPs with conventional antibiotics can possibly be researched as alternatives to antimicrobial agents for curing infections caused by MDR *Pseudomonas aeruginosa* following multiple clinical trials.

## Supplementary Information

Supplementary Information.

## Data Availability

All data generated or analysed during this study are included in this published article (and its Supplementary Information file).

## Abbreviations

MBL: Metallo β-lactamase
ESBL: Extended spectrum β-lactamase
GEN: Gentamicin
MEM: Meropenem
IMP: Imipenem
SCF: Cefoperazone-Sulbactam
TZP: Piperacillin/Tazobactam
CIP: Ciprofloxacin
AZT: Aztreonam
CAZ: Ceftazidime
AMK: Amikacin
CFX: Cefoxitin
UV: Ultra-violet
LB: Luria Bertani
MH: Mueller–hinton
ATCC: American Type Cell Culture
MAR index: Multiple antibiotic resistance index
MIC: Minimum inhibitory concentration
CLSI: Clinical & Laboratory Standards Institute
MHT: Modified Hodge Test
CDST: Combined Disc Synergy Test
AgNPs: Silver nanoparticles
WHO: World Health Organization
LMIC: Low income and middle income countries
MDR-PA: Multidrug resistant *Pseudomonas aeruginosa*
CDC: Centers for Disease Control and Prevention
