# Supplementary material for: Antibacterial efficacy of silver nanoparticles (AgNPs) against metallo-β-lactamase and extended spectrum β-lactamase producing clinically procured isolates of Pseudomonas aeruginosa
Source: Sci Rep. 2022 Nov 30;12:20685. doi: 10.1038/s41598-022-24531-9 (PMC9712613; doi:10.1038/s41598-022-24531-9)
Supplement: Supplementary file 1 — Supplementary Information. [file 41598_2022_24531_MOESM1_ESM.pdf]

Title: Antibacterial efficacy of silver nanoparticles (AgNPs) against metallo-β-lactamase and extended spectrum β-lactamase producing clinically procured isolates of *Pseudomonas aeruginosa*

Running Title: Antibacterial efficacy of AgNPs metallo-β-lactamase extended spectrum β-lactamase *Pseudomonas aeruginosa*

Authors Order

Maria Muddassir[1], Almas Raza[1], Sadaf Munir[2], Ahmad Basirat[3], Muddassir Ahmed[4], Mazia Shahid Butt[5], Omair Arshad Dar[6] ,Syed Shoaib Ahmed[7], Saba Shamim[1], Syed Zeeshan Haider Naqvi[1 *]

[1]Institute of Molecular Biology & Biotechnology (IMBB), The University of Lahore. Defence Road Campus, Lahore. Pakistan

[2] Combined Military Hospital, Lahore. Pakistan

[3] King Edward Medical University, Lahore. Pakistan.

[4] Social Security Hospital, Gujranwala. Pakistan

[5] Lahore General Hospital, Lahore. Pakistan

[6] Pak Emirates Military Hospital, Rawalpindi. Pakistan

[7] Al-Aleem Centre for Advanced Studies & Research, Gulab Devi Educational Complex, Lahore. Pakistan

[5] Al-Aleem Centre for Advanced Studies & Research, Gulab Devi Educational Complex, Lahore. Pakistan

Corresponding author:

Dr. Syed Zeeshan Haider

(Ph. D Microbiology)

Research Supervisor & Principle Investigator

Institute of Molecular Biology & Biotechnology (IMBB), The University of Lahore, Pakistan.

zeeshan.haider@imbb.uol.edu.pk

Supplementary Table 1 (ST_1) PCR conditions of individual resistance genes

Each step of PCR	PCR condition (°C, minutes, seconds)					
	bla$_{\mathbf{IMP-1}}$	***bla***$_{\mathbf{VIM}}$	***bla***$_{\mathbf{TEM}}$	***bla***$_{\mathbf{SHV}}$	***bla***$_{\mathbf{OXA}}$	***bla***$_{\mathbf{AmpC}}$
**Predenaturing**	95°C<br>10 min	95°C<br>10 min	95°C<br>10 min	95°C<br>10 min	95°C<br>10 min	95°C<br>10 min
35 cycles of						
**Denaturing**	95°C<br>20 sec	95°C<br>20 sec	95°C<br>20 sec	95°C<br>20 sec	95°C<br>20 sec	95°C<br>20 sec
**Annealing**	53°C<br>30 sec	57°C<br>30 sec	55°C<br>30 sec	55°C<br>30 sec	55°C<br>30 sec	65°C<br>30 sec
**Extension**	72°C<br>30 sec	72°C<br>30 sec	72°C<br>30 sec	72°C<br>30 sec	72°C<br>30 sec	72°C<br>30 sec
**Plate read**						
**Final Extension**	72°C<br>5 min	72°C<br>5 min	72°C<br>5 min	72°C<br>5 min	72°C<br>5 min	72°C<br>5 min
**Melting curve 65°C to 95°C, increment 5°C for 0:05 + Plate read**						
